# Interpretation of dynamic tensile behavior by austenite stability in ferrite-austenite duplex lightweight steels

**DOI:** 10.1038/s41598-017-15991-5

**Published:** 2017-11-16

**Authors:** Jaeyeong Park, Min Cheol Jo, Hyeok Jae Jeong, Seok Su Sohn, Jai-Hyun Kwak, Hyoung Seop Kim, Sunghak Lee

**Affiliations:** 1 Center for Advanced Aerospace MaterialsPohang University of Science and Technology, Pohang, 790-784 Republic of Korea; 2 Max-Planck-Institut für Eisenforschung Max-Plank-Straβe 1, Düsseldorf, 40237 Germany; 3 Sheet Products & Process Research Group Technical Research Laboratories, POSCO, Kwangyang, 545-090 Republic of Korea

## Abstract

Phenomena occurring in duplex lightweight steels under dynamic loading are hardly investigated, although its understanding is essentially needed in applications of automotive steels. In this study, quasi-static and dynamic tensile properties of duplex lightweight steels were investigated by focusing on how TRIP and TWIP mechanisms were varied under the quasi-static and dynamic loading conditions. As the annealing temperature increased, the grain size and volume fraction of austenite increased, thereby gradually decreasing austenite stability. The strain-hardening rate curves displayed a multiple-stage strain-hardening behavior, which was closely related with deformation mechanisms. Under the dynamic loading, the temperature rise due to adiabatic heating raised the austenite stability, which resulted in the reduction in the TRIP amount. Though the 950 °C-annealed specimen having the lowest austenite stability showed the very low ductility and strength under the quasi-static loading, it exhibited the tensile elongation up to 54% as well as high strain-hardening rate and tensile strength (1038 MPa) due to appropriate austenite stability under dynamic loading. Since dynamic properties of the present duplex lightweight steels show the excellent strength-ductility combination as well as continuously high strain hardening, they can be sufficiently applied to automotive steel sheets demanded for stronger vehicle bodies and safety enhancement.

## Introduction

Since automotive steels generally require excellent strength for sustaining structures and improving resistances to shock or impact during car crashes^1–3^, highly-deformable TWinning Induced Plasticity (TWIP) and TRansformation Induced Plasticity (TRIP) steels have been used in automotive industries. Beside of strict strength and ductility requirements, reduction in vehicle’s weight and environmental friendliness are key issues in automotive applications^4–6^. In order to achieve the lightweight effect, thus, Al-containing (α + γ) duplex lightweight steels have been actively developed as a realistic measure^7–9^. The addition of 5~6 wt.% of Al as well as 8~9 wt.% of Mn provides excellent tensile properties including tensile strength above 700 MPa and elongation above 70%^10^, while it also leads to about 9% of lightweight effect compared to conventional steels^11^. This excellent strength-ductility combination is mainly achieved at an optimal austenite stability obtained when both TWIP and TRIP mechanisms are actively working^10^.

Phenomena occurring in duplex lightweight steels under dynamically loaded conditions are hardly investigated, although phenomena under quasi-statically loaded conditions have been well studied^12,13^. To effectively use duplex lightweight steels for automotive steel sheet applications, particularly for collision-absorption components, the detailed information of dynamic deformation should be obtained. This is because automotive steel sheets require high resistance to impact energy upon vehicle collision as well as high strength and fracture toughness for sustaining sufficient structural stability. Accurate evaluation should also be performed on how safe the steel sheets are under worst conditions like vehicle collision. In vehicle-collision tests, actual vehicles are generally used^14^, but restrictions remain in applying the evaluation data to the vehicle body safety itself and in styling new car designs. Accordingly, the vehicle body safety data are essentially needed before the evaluation of actual vehicle products.

In general, mechanical properties and deformation behavior under the dynamic loading are explained mainly by the stacking fault energy (SFE) of austenite^15–17^. It has been known that this SFE decreases as the temperature decreases or the strain rate increases^17,18^. Under the dynamic loading, the time for the heat to be emitted outside is not enough because the deformation rapidly occurs within several hundreds of microseconds, which usually induces an adiabatic heating to raise the temperature inside the material^19^. Effects of strain rate and temperature on deformation mechanisms have been explained differently by materials or researchers^16,20–23^. Lee *et al*.^20^ reported that the transformation amount of α’-martensite increased with increasing strain rate in 304 L stainless steels by the following two reasons: (1) well-developed shear bands due to more favorable formation and movement of Shockley partial dislocations than perfect dislocations^21^, and (2) active martensitic transformation at single shear band itself as well as intersections of shear bands^20^. On the other hand, Talonen *et al*.^22^ confirmed the decrease in martensitic transformation with increasing strain rate in an austenitic stainless steel because the shear band formation was reduced by the increase in SFE due to the temperature rise.

The deformation twinning behavior is also affected by the strain rate. Lee *et al*.^16^ argued that the twinning was gradually activated as the strain rate increased because the rate sensitivity of critical resolved shear stress for TWIP was much lower than that for slip or TRIP. According to Liang *et al*.^23^, however, the dislocation glide was more activated than the deformation twinning by the increase in SFE due to adiabatic heating, which resulted in the decrease in strain hardening. The strain rate hardening effect and temperature effect act on TRIP and TWIP competitively under the dynamic deformation of the austenite. Thus, which effect predominates or overrides plays an important role in determining the deformation behavior, but the detailed dynamic deformation behavior including both TWIP and TRIP is hardly studied in automotive steels, particularly in duplex lightweight steels.

In this study, (α + γ) duplex lightweight steel microstructures showing both TWIP and TRIP mechanisms were designed by annealing an Fe-0.3C-8.8Mn-5.1Al steel. Dynamic and quasi-static tensile properties were measured under strain rates of about 3000 sec^−1^ and 10^−3^ sec^−1^ by using a split Hopkinson tensile bar and a universal testing machine, respectively, instead of various ranges of strain rate^15,16^. This comparison of dynamic and quasi-static tensile properties would be sufficiently meaningful for investigating the dynamic mechanical behavior. Detailed deformation mechanisms were also examined by analyzing how TWIP and TRIP mechanisms were varied under the quasi-static and dynamic loadings, and the correlation with microstructural evolution processes was verified.

## Results

### Microstructures of duplex lightweight steel specimens

**Figure 1:**
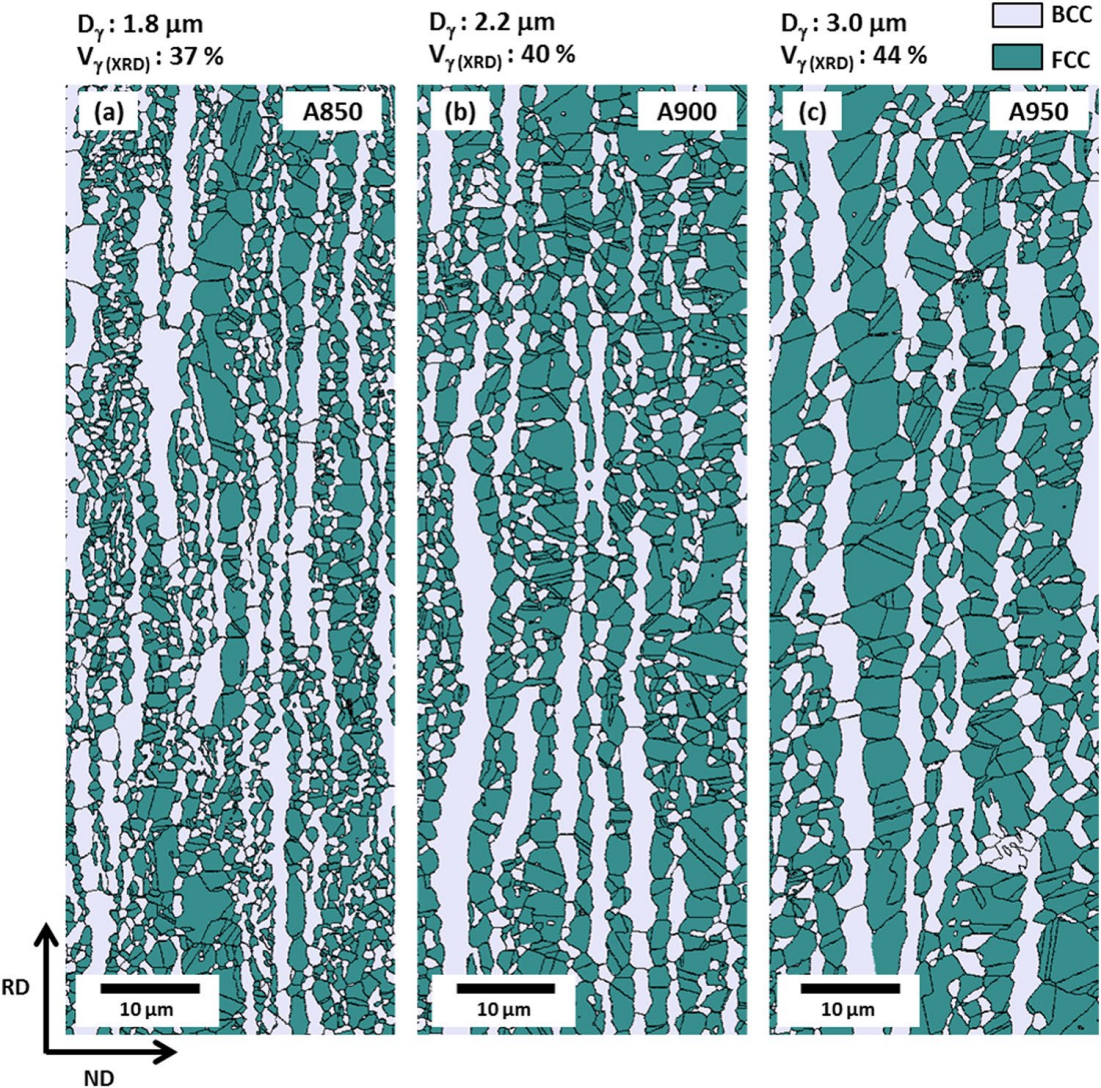
EBSD phase maps of the (**a**) A850, (**b**) A900, and (**c**) A950 specimens. All the annealed specimens consist of ferrite and austenite. The volume fraction of austenite (V_γ_) and grain size of austenite (D_γ_) are indicated above each map.

EBSD phase maps of the A850, A900, and A950 specimens are shown in Fig. 1(a–c). All the annealed specimens consist of ferrite and austenite, and exhibit band microstructures elongated along the rolling direction. The volume fraction (V_γ_) and grain size (D_γ_) of austenite were measured, and are indicated above each map. The austenite volume fraction and grain size of the A850 specimen are 37% and 1.8 μm, respectively (Fig. 1(a)), and increase to 44% and 3.0 μm, respectively, in the A950 specimen (Fig. 1(c)). A number of annealing twins are found inside austenite grains.

### Room-temperature quasi-static and dynamic tensile properties

**Figure 2:**
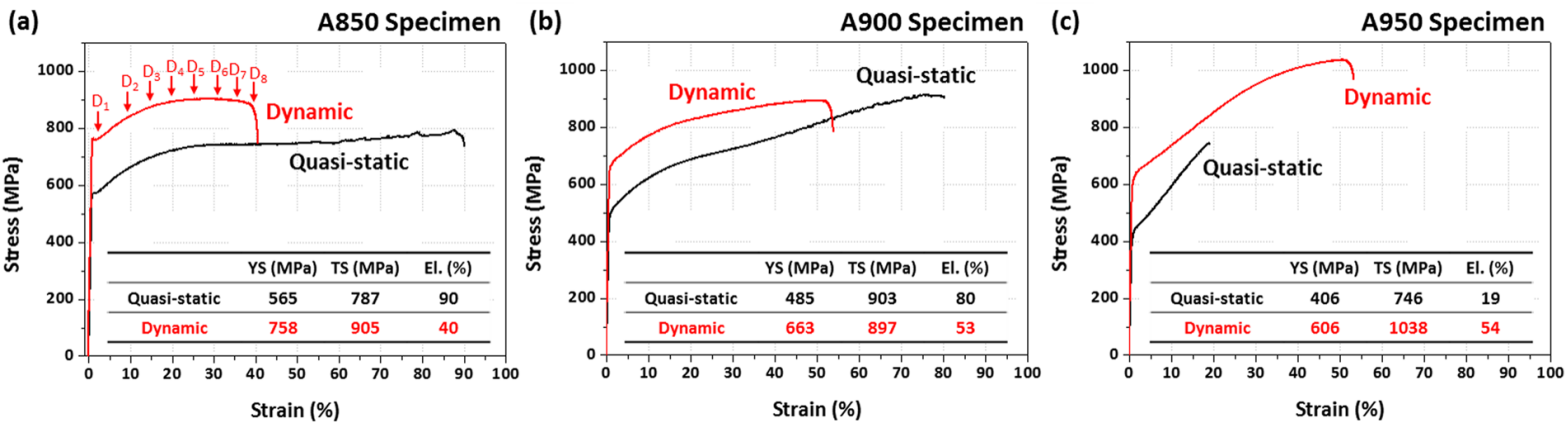
Room-temperature engineering quasi-static and dynamic stress-strain curves of the (**a**) A850, (**b**) A900, and (**c**) A950 specimens. It is noted that the dynamic strengths and elongation are higher than the quasi-static ones in the A950 specimen.

Room-temperature engineering quasi-static and dynamic stress-strain curves of the annealed specimens are shown in Fig. 2(a–c), from which tensile properties are measured, as summarized in Table 1. In the case of the dynamic tensile test, incident, reflective, and transmitted waves were obtained from the split Hopkinson tensile bar, as shown in Supplementary Fig. S1, from which dynamic stress-strain curves were drawn.

**Table 1:** Room-temperature quasi-static and dynamic tensile properties of the A850, A900, and A950 steel specimens.

Specimen	Quasi-static			Dynamic		
	Yield Strength (MPa)	Tensile Strength (MPa)	Total Elongation (%)	Yield Strength (MPa)	Tensile Strength (MPa)	Total Elongation (%)
A850	565 ± 3	787 ± 8	90 ± 4	758 ± 7	905 ± 9	40 ± 3
A900	485 ± 5	903 ± 11	80 ± 3	663 ± 7	897 ± 10	53 ± 4
A950	406 ± 4	746 ± 12	19 ± 3	606 ± 6	1038 ± 12	54 ± 3

In order to investigate the detailed deformation behavior and to confirm the uniformity of the dynamically tensioned specimen, the vision strain gauge system was simultaneously utilized during the dynamic tensile test. Eight digital images of strain distributions of the dynamically tensioned A850 specimen were obtained from the system, as shown in Supplementary Fig. S2a. They are numbered by ‘D_1_’ through ‘D_8_’, which are also marked by arrows in the dynamic stress-strain curve of Fig. 2a by matching the digital image photographing time with the wave-signal recording time in the oscilloscope. Here in the digital images, the strain distributions are indicated by blue, green, yellow, and red colors, based on the strain amount. Until the deformation proceeds to the D_5_ stage at the time of 86 μs, the strain continuously increases up to 25.2%, but the deformation is uniform throughout the gage section. In the D_6_ stage near the tensile instability (necking) point, the deformation becomes slightly non-uniform, and the maximum local strain is 37.5%. In the final D_7_ and D_8_ stages before the fracture (115~129 μs), the highly strained region (yellow- or red-colored region) appears in the center of the gage section. The local strains calculated from digital images along the center line as a function of distance from the bottom of the gage section (gage length; 7 mm) are plotted in Supplementary Fig. S2b. In the D_1_~D_5_ stages, local strain distribution curves are almost flat, which shows the uniform deformation behavior. The A900 and A950 specimens also show the uniform dynamic deformation behavior in the initial and intermediate deformation stages.

The quasi-static yield strength, tensile strength, and elongation of the A850 specimen are 565 MPa, 787 MPa, and 90%, respectively. The tensile stress almost maintains or very slightly increases from the strain of 30% to the failure strain (90%), and shows a serrated flow from the strain of 50% (Fig. 2(a)). The A900 specimen shows the lower yield strength, higher tensile strength, lower elongation, and higher strain hardening than the A850 specimen (Fig. 2(b)). Both the A850 and A900 specimens exhibit an excellent combination of tensile strength (above 780 MPa) and elongation (above 80%). The A950 specimen shows the lowest tensile properties because it fractures at the lowest strain (19%) without plastic instability (Fig. 2(c)). As the annealing temperature increases in the three specimens, the yield strength and elongation decrease, while the strain-hardening rate increases.

The dynamic yield strength, tensile strength, and elongation of the A850 specimen are 758 MPa, 905 MPa, and 40%, respectively (Fig. 2(a)). The strengths increase by the strain rate hardening effect^24^, while the elongation drops, in comparison with quasi-static properties. The failure occurs after the tensile instability, unlike in the quasi-static case. In the A900 specimen, the strain rate hardening effect, *i.e*., strength increase and elongation decrease, also appears, but the decreased amount in elongation is smaller than that of the A850 specimen (Fig. 2(b)). In the A950 specimen, both the dynamic strengths and elongation are higher than the quasi-static ones (Fig. 2(c)). It is also noted that the dynamic elongation is highest among the specimens. The tensile strength and elongation tend to increase with increasing annealing temperature, while the yield strength decreases.

### Tensile deformation mechanisms

**Figure 3:**
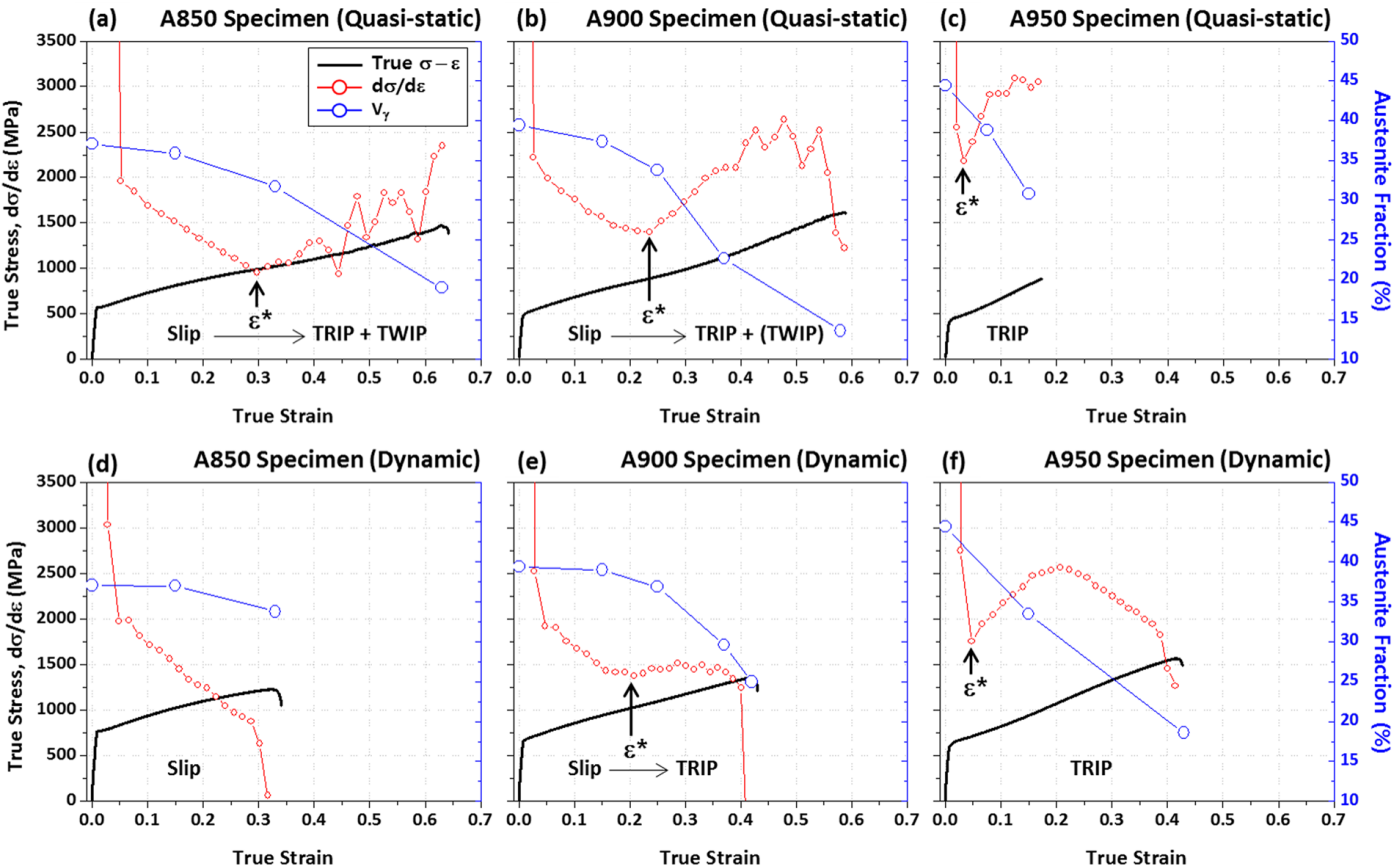
Room-temperature true quasi-static and dynamic stress-strain curves, strain-hardening rate (dσ/dε) curves, and volume fractions of austenite measured by the direct comparison XRD method for the (**a,d**) A850, (**b,e**) A900, and (**c,f**) A950 specimens. The strain-hardening rate curves display a multiple-stage strain-hardening behavior, *e.g*., falling and rising stages, and the true strain at the inflection point is denoted by ‘ε*’.

True quasi-static and dynamic stress-strain curves, strain-hardening rate (dσ/dε) curves, and austenite volume fractions (measured from the XRD method^25^) are shown in Fig. 3(a–f). The strain-hardening rate curves display a multiple-stage strain-hardening behavior, *e.g*., falling and rising stages, and the true strain at the inflection point is denoted by ‘ε*’. Under the quasi-static loading, the strain-hardening rate decreases until the true strain of 0.3, and increases with a serrated flow behavior in the A850 specimen, which indicates that the ε* is 0.3 (Fig. 3(a)). As the annealing temperature increases to 900 °C and 950 °C, the ε* decreases to 0.23 and 0.02, respectively, while overall strain-hardening rates tend to increase (Fig. 3(b,c)). The A950 specimen shows a very high strain-hardening rate without plastic instability, thereby leading to the rapid failure. Under the dynamic loading, the strain-hardening rate decreases continuously in the A850 specimen, and thus the ε* cannot be defined (Fig. 3(d)). The ε* of the A900 specimen (0.2) is similar to that under the quasi-static loading, but the strain-hardening rate remains almost steadily after the ε* (Fig. 3(e)). The ε* of the A950 specimen (0.03) is also similar to that under the quasi-static loading (Fig. 3(f)). It is noted that the plastic instability occurs under the dynamic loading, unlike under quasi-static loading.

The austenite volume fraction (Vγ) decreases with increasing true strain in the three specimens, which implies the occurrence of martensitic transformation during the deformation. In the A850 specimen, the austenite fraction reduces rather rapidly from the ε* (0.3) under the quasi-static loading, whereas it does not much decrease under the dynamic loading (Fig. 3(a,d)). In the A900 specimen, the austenite fraction reduces rapidly from the ε* (about 0.2) under both the quasi-static and dynamic loadings (Fig. 3(b,e)), and its reduced amount is larger under the quasi-static loading. In the A950 specimen whose ε* is very low below 0.05, the austenite fraction decreases rapidly at low true strain levels under both the quasi-static and dynamic loadings (Fig. 3(c,f)). Under the dynamic loading, the TRIP occurs continuously up to the true strain of 0.4, which raises the elongation.

**Figure 4:**
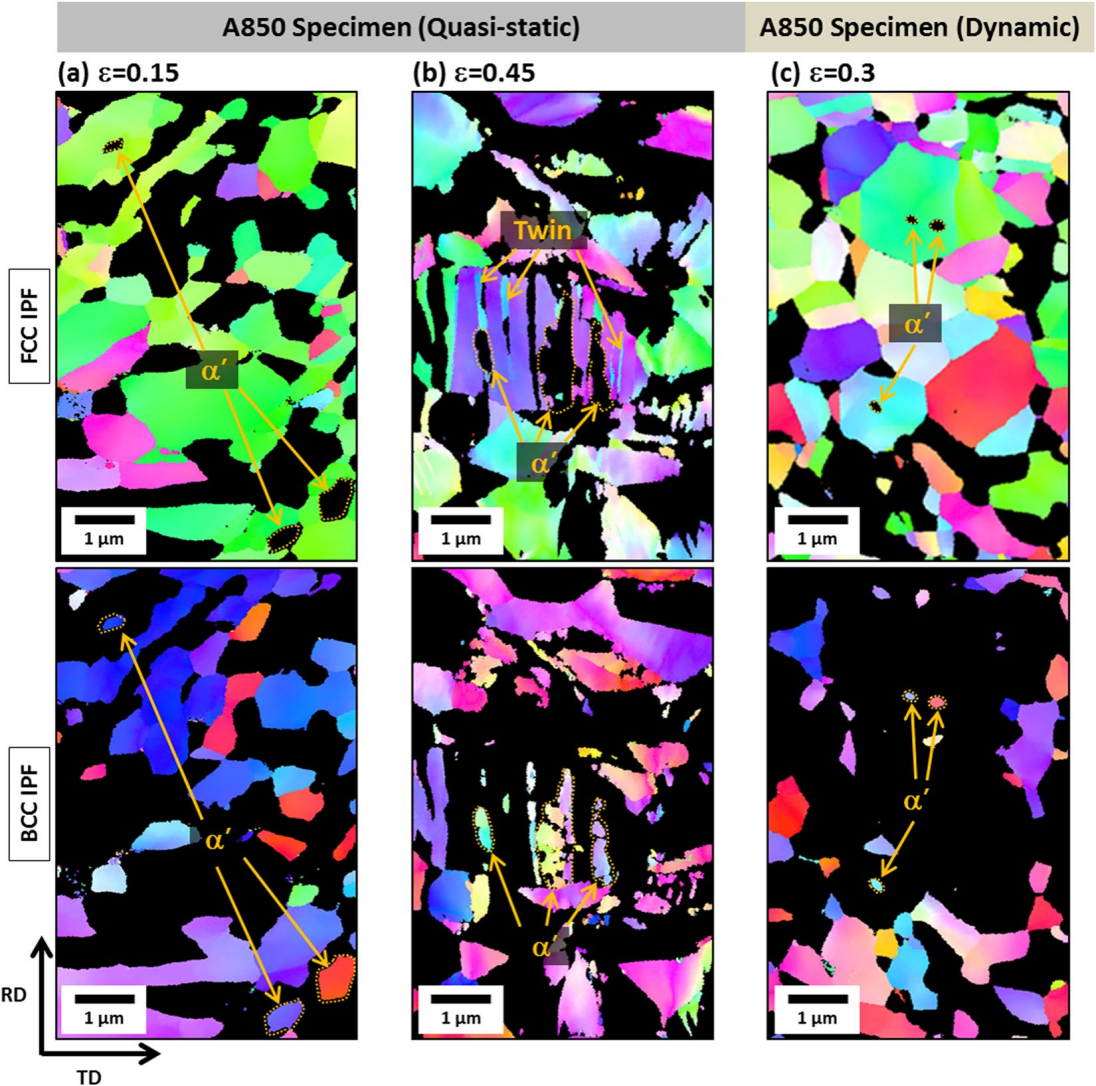
EBSD FCC and BCC inverse pole figure (IPF) maps of the A850 specimen. (**a,b**) At the true strains of 0.15 and 0.45 under the quasi-static loading, and (**c**) at the true strain of 0.3 under the dynamic loading. Under the quasi-static loading, the major deformation mechanism is the dislocation slip before the ε*, both TRIP and TWIP after the ε*. Under the dynamic loading, the dislocation slip is the major mechanism.

**Figure 5:**
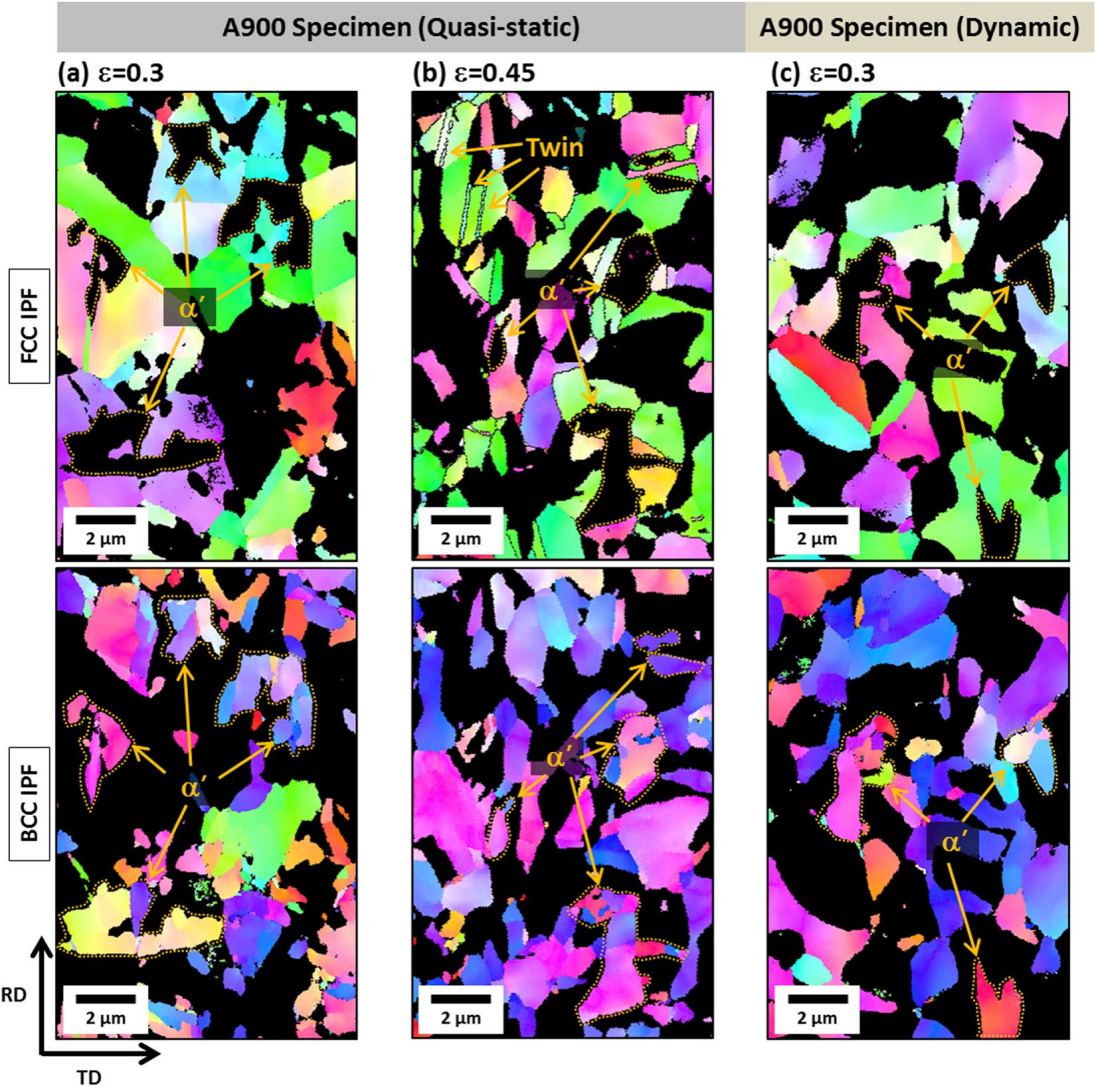
EBSD FCC and BCC inverse pole figure (IPF) maps of the A900 specimen (**a,b**) at the true strains of 0.3 and 0.45 under the quasi-static loading, and (**c**) at the true strain of 0.3 under the dynamic loading. Under the quasi-static loading, the TRIP is the major deformation mechanism and a few deformation twins are observed. Under the dynamic loading, the major deformation mechanism is the TRIP, but the amount of TRIP is smaller than that of the quasi-static case.

**Figure 6:**
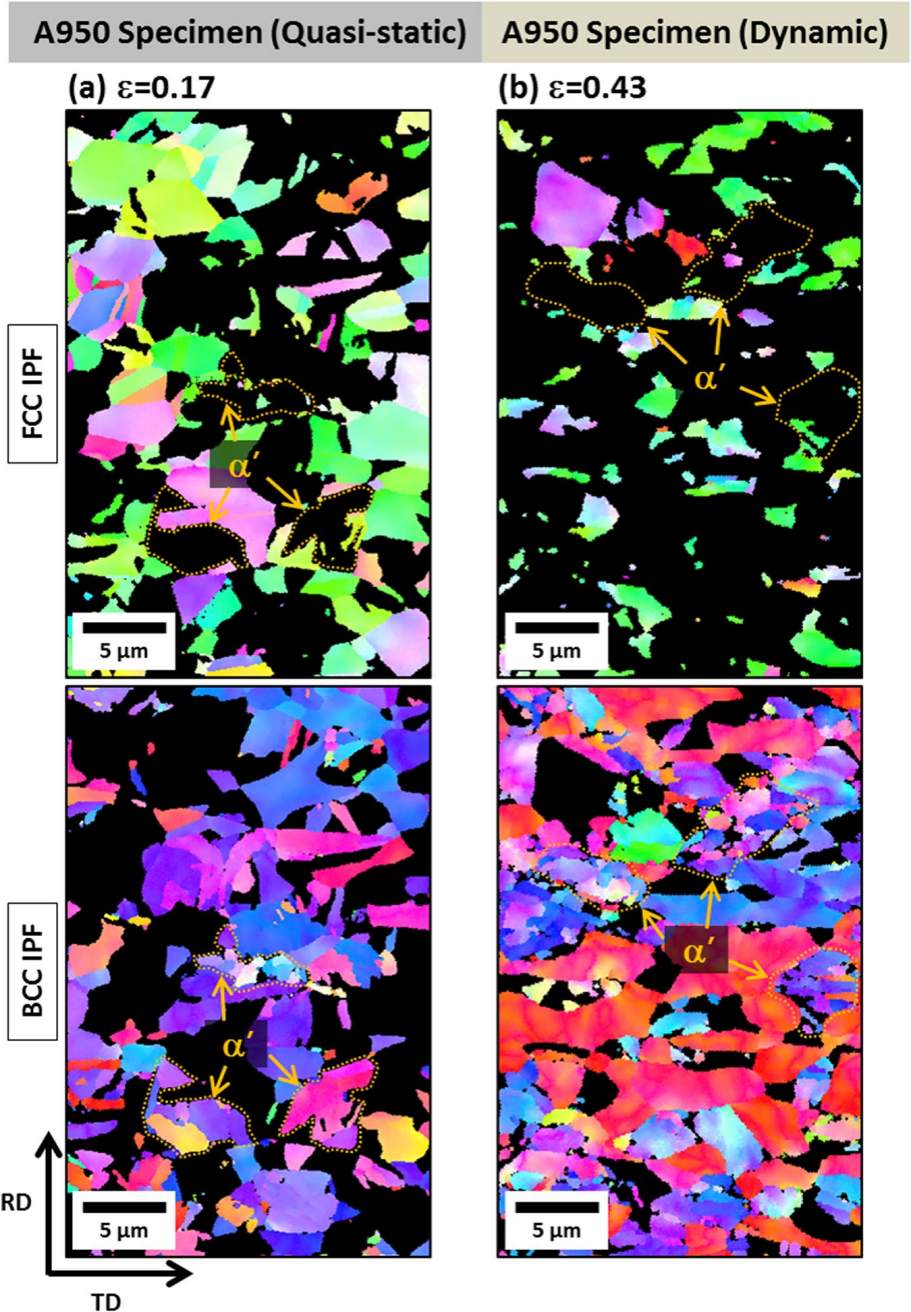
EBSD FCC and BCC inverse pole figure (IPF) maps of the A950 specimen (**a**) at the true strain of 0.17 under the quasi-static loading and (**b**) at the true strain of 0.43 under the dynamic loading. Under the quasi-static loading, a considerable amount of TRIP is observed throughout many austenite grains. Under the dynamic loading, the amount of TRIP is larger than that of the quasi-static case.

The transformation rate as well as strain-hardening rate are varied with annealing temperature and strain rate change, which is also affected by deformation mechanisms. The EBSD analyses were conducted to examine the detailed deformation behavior in each strain-hardening stage. Figures 4–6 shows FCC and BCC inverse pole figure (IPF) maps of the quasi-statically or dynamically tensioned A850, A900, and A950 specimens, respectively, at different true strains. Since the dynamically tensioned specimen is rapidly fractured, the interrupted dynamic tensile test was performed after a newly designed dynamic tensile specimen was placed in an external interruption device^26^. In this interrupted test, basic assumptions of the split Hopkinson tensile bar test, *i.e*., uniform strain in the specimen and one-dimensional wave, have been violated^27^, and dynamic stress-strain curves could be problematic because this problem might be unsolvable owing to the nature of dynamic tests. Thus, the interrupted dynamic tensile test is useful only for the microstructural observation purpose at different dynamic true strains. Under the quasi-static loading of the A850 specimen, the TRIP slightly occurs at the true strain of 0.15 and twins are not found (Fig. 4(a)). This implies that the major mechanism is the dislocation slip before the ε* (0.3). At the true strain of 0.45 (after the ε*), both α’-martensite and deformation twins are formed inside austenite grains, as indicated by arrows. (Fig. 4(b)). This indicates that the increased strain-hardening rate in the A850 specimen is associated with the simultaneous occurrence of TRIP and TWIP. Under the dynamic loading, both TRIP and TWIP hardly occur at the true strain of 0.3 (Fig. 4(c)), like at the true strain of 0.15 under the quasi-static loading (Fig. 4(a)). This indicates that the dynamically tensioned A850 specimen is mainly deformed by the dislocation slip. Under the dynamic loading, the TRIP and TWIP are prevented, which results in the insufficient strain hardening and consequent low elongation (40%).

Under the quasi-static loading of the A900 specimen, the TRIP mainly occurs at the true strain of 0.3 (after the ε* (0.23)) where the austenite fraction decreases considerably rapidly, and deformation twins are hardly found (Fig. 5(a)). At the higher true strain of 0.45, the TRIP occurs steadily, and deformation twins are observed inside some austenite grains, although their number is much smaller than that of the A850 specimen (Fig. 5(b)). The major deformation mechanism after the ε* is the TRIP, unlike in the A850 specimen, which results in the increased strain-hardening rate (Fig. 3(b)). Under the dynamic loading at the true strain of 0.3 (after the ε* (0.2)), the major deformation mechanism is the TRIP (Fig. 5(c)), but the amount of TRIP is smaller than that of the quasi-static case (Fig. 5(a)) which is also confirmed from Fig. 3(b,e) (11% *vs*. 6%). Under the dynamic loading, the TRIP is suppressed and TWIP is prevented, which results in the insufficient strain hardening and consequent low elongation (53%).

Under the quasi-static loading of the A950 specimen, a considerable amount of TRIP is observed throughout many austenite grains at the failure strain of 0.17 (after the ε* (0.02)) (Fig. 6(a)). Under the dynamic loading at the failure strain of 0.43 (after the ε* (0.03)), the TRIP occurs actively (Fig. 6(b)). This active TRIP leads to the high strain hardening (Fig. 3(f)) because about 25 vol.% of austenite is reduced by the steady TRIP until the failure. According to Song *et al*.^28^, when duplex lightweight steels having low austenite stability were deformed, the strain-hardening rate was raised by the rapid TRIP, but the additional TRIP became difficult. In this rapid TRIP case, small voids or cracks initiate at martensite/ferrite interfaces or martensite itself at low strain levels, and propagate across ferrite grains, which results in the low ductility together with brittle fracture. That is, the strain localization in a shallow area followed by the rapid failure occurs, and leads to the low ductility. Deformation twins are not formed under the quasi-static and dynamic loadings. When the deformation behaviors of the three annealed specimens are summarized, the martensitic transformation rate increases with increasing annealing temperature because the stability of austenite decreases, while the ε* decreases. On the contrary, the stability of austenite tends to increase under the dynamic loading, while the ε* is not varied much. This result implies that the strain required for TRIP is similar under both the quasi-static and dynamic loadings, but the transformation rate is lower under the dynamic loading.

## Discussion

### Austenite stability varied with annealing temperature

The SFE of austenite, which is varied with chemical composition, temperature, and grain size of austenite, affects the deformation behavior^17,29^. It is well known that deformation mechanisms in austenite change from wavy slip to Microband-, TWinning-, and TRansformation-Induced Plasticity (MBIP, TWIP, and TRIP, respectively) as the SFE decreases^30^. The present (α + γ) duplex lightweight steel specimens contain a considerable amount of Al, a ferrite stabilizer. Since Al decreases the austenite stability against martensitic transformation and increases the SFE^29,31^, the SFE is not the only parameter determining deformation mechanisms of austenite in the duplex lightweight steel specimens.

The austenite stability is simply expressed by athermal martensite start temperature (M_S_) which means the austenite to martensite transformation temperature without any deformation^32^. The M_S_ was calculated by using the following equations^32,33^:

1 $${{\rm{M}}}_{{\rm{S0}}}(^\circ {\rm{C}})=539-423{\rm{C}}-30.4{\rm{Mn}}-{\rm{7}}{\rm{.5Si}}+30{\rm{Al}}$$

2 $${{\rm{M}}}_{{\rm{S}}}(^\circ {\rm{C}})={{\rm{M}}}_{{\rm{S0}}}-{{{\rm{B}}({\rm{V}}}_{\gamma }}^{-1/3})$$

where Ms_0_, V_γ_, and B show the effect of alloy composition on M_S_, average austenite volume, and geometrical coefficient of grain (475 μmK), respectively. The SFE (Γ) was calculated by a thermodynamic model as follow^17,34^:

3 $$\begin{array}{rcl}{\rm{\Gamma }} & = & 2{\rm{\rho }}{\rm{\Delta }}{{\rm{G}}}^{{\rm{\gamma }}\to {\rm{\varepsilon }}}+2{{\rm{\sigma }}}^{{\rm{\gamma }}/{\rm{\varepsilon }}}+2{\rm{\rho }}{\rm{\Delta }}{{\rm{G}}}_{{\rm{e}}{\rm{x}}}\\ {\rm{\Delta }}{{\rm{G}}}^{{\rm{\gamma }}\to {\rm{\varepsilon }}} & = & {{\rm{\chi }}}_{{\rm{F}}{\rm{e}}}{\rm{\Delta }}{{\rm{G}}}_{{\rm{F}}{\rm{e}}}+{{\rm{\chi }}}_{{\rm{M}}{\rm{n}}}{\rm{\Delta }}{{\rm{G}}}_{{\rm{M}}{\rm{n}}}+{{\rm{\chi }}}_{{\rm{A}}{\rm{l}}}{\rm{\Delta }}{{\rm{G}}}_{{\rm{A}}{\rm{l}}}+{{\rm{\chi }}}_{{\rm{C}}}{\rm{\Delta }}{{\rm{G}}}_{{\rm{F}}{\rm{e}}{\rm{M}}{\rm{n}}{\rm{X}}/{\rm{C}}}\\  &  & +\,{{\rm{\chi }}}_{{\rm{F}}{\rm{e}}}{{\rm{\chi }}}_{{\rm{M}}{\rm{n}}}{\rm{\Delta }}{{\rm{\Omega }}}_{{\rm{F}}{\rm{e}}{\rm{M}}{\rm{n}}}+{{\rm{\chi }}}_{{\rm{F}}{\rm{e}}}{{\rm{\chi }}}_{{\rm{A}}{\rm{l}}}{\rm{\Delta }}{{\rm{\Omega }}}_{{\rm{F}}{\rm{e}}{\rm{A}}{\rm{l}}}+{\rm{\Delta }}{{\rm{G}}}_{{\rm{m}}{\rm{g}}}\\ {\rm{\Delta }}{{\rm{G}}}_{{\rm{e}}{\rm{x}}} & = & 170.06{\rm{e}}{\rm{x}}{\rm{p}}(-{\rm{d}}/18.55)\end{array}$$

where ρ, ΔG^γ→ε^, σ^γ/ε^, ΔG_ex_, χ_ϕ_, ΔG_ϕ_, ΔΩ_Feϕ_, and ΔG_mg_ are the molar surface density on {111} planes, change in molar Gibbs free energy change for the γ→ε phase transformation in relation with chemical compositions, γ/ε interfacial energy, excess SFE due to effect of grain size (d), molar fractions of pure alloying elements, change in molar Gibbs energy upon the γ→ε phase transformation for pure elements ϕ, excess free energy caused by interaction between Fe and other elements ϕ, and magnetic contribution, respectively. ΔG_ϕ_, ΔΩ_Fe_ϕ, and ΔG_mg_ are calculated from empiric formulas in references^29,35,36^. The ΔG^γ→ε^ term contains the temperature variation. The SFE is decreased by the increased austenite grain size according to Eq. (3). C, Mn, and Al contents measured from the XRD and EPMA analyses, austenite grain size, and calculated M_S_ temperature, and SFE of the three specimens are summarized in Table 2.

**Table 2:** C, Mn, and Al contents in austenite grain, austenite grain size, M_S_ temperature, and SFE of the A850, A900, and A950 specimens.

Specimen	C^*^ (wt.%)	Mn^**^ (wt.%)	Al^**^ (wt.%)	Grain Size (μm)	M_S0_^***^ (°C)	M_S_^***^ (°C)	SFE^***^ (mJ/m^2^)
A850	0.57	11.61	4.46	1.8	79	−268	50.4
A900	0.55	11.25	4.53	2.2	100	−180	49.5
A950	0.49	10.63	4.60	3.0	147	−80	46.5

*Content of C measured from the XRD analysis.

**Contents of Mn and Al measured from the EPMA analysis.

***M_S_ temperatures and SFE calculated from Eqs (1) through (3).

In the duplex microstructures, C and Mn are readily partitioned into austenite grains, whereas Al is mostly partitioned into ferrite grains^37^. Since the austenite fraction increases with increasing annealing temperature (Fig. 1(a–c)), smaller amounts of C and Mn exist in austenite grains, which results in the lower austenite stability, as shown in Table 2. Furthermore, austenite grains are coarsened with increasing annealing temperature, thereby leading to the increased M_S_, which implies the decrease in austenite stability according to Eq. (2). The M_S_ is lowest (−268 °C) in the A850 specimen whose austenite fraction and grain size are smallest, and is highest (−80 °C) in the A950 specimen whose austenite fraction and grain size are largest. Consequently, the austenite stability decreases with increasing annealing temperature, and a smaller amount of deformation is needed for the martensitic transformation. This is related with the reduction in ε* in Fig. 3(a–f). Since the deformation amount required for the martensitic transformation decreases with decreasing austenite stability, the austenite fraction reduces rapidly, and the strain-hardening rate increases.

### Effects of strain rate hardening on austenite stability

Under the dynamic loading, the yield strength increases by about 200 MPa in the three specimens (Fig. 2(a–c)) according to the strain rate hardening effect^24^. This strain rate hardening is associated with the following two reasons. Firstly, the dislocation mobility decreases because the dislocation movement mechanism changes from thermally activated behavior to viscous behavior with increasing strain rate^38,39^. The plastic deformation is accommodated by the dislocation movement, and the dislocation mobility decreases at the higher strain rate. In order to accommodate the plastic deformation, thus, the dislocation density should greatly increase^40,41^. The second reason is a shape change of dislocations. According to Huh *et al*.^42^, the dislocation shape changes from a round half-loop shape to a straight shape as the strain rate increases, which promotes the formation of entangled dislocations. As a result, entangled dislocations are populated by the increase in dislocation density and dislocation shape change with increasing strain rate, and the flow stress required for slip increases, thereby resulting in the increase in yield strength.

As well as the yield strength increase, the austenite to martensite transformation is suppressed under the dynamic loading (Fig. 3(a–f)). In conventional austenite-containing steels, it is well known that the TRIP and TWIP mechanisms affect importantly tensile properties. In the present duplex lightweight steel specimens, both the TRIP and TWIP mechanisms occur only in the A850 specimen under the quasi-static loading, and the TWIP mechanism is hardly found in other cases, as shown in Figs 4–6. This indicates that the TRIP mechanism is more importantly working overall in the present lightweight steel specimens under the quasi-static and dynamic loadings than the TWIP mechanism.

In order to reveal the interaction between strain rate and martensitic transformation rate, the transformation rate varied with the annealing temperature and strain rate has been quantitatively analyzed. Olson and Cohen^43^ proposed the following equation based on a nucleation and growth mechanism of strain-induced martensitic transformation:

4 $${{\rm{f}}}_{{\alpha }^{\mbox{'}}}={\rm{1}}\,-\exp \{-\beta {[1-\exp (-\alpha \varepsilon )]}^{{\rm{p}}}\}$$

where f_α’_ and ε are volume fraction of α’-martensite and true strain, respectively. The p is an exponent of 4.5, which means the intersecting probability of a shear band with another shear band. The α parameter represents the formation rate of shear bands which act as nucleation sites of α’-martensite embryos at a given strain, and the β parameter indicates the growth probability of α’-martensite embryo at a given shear band intersection. Thus, α and β parameters are closely related with the SFE and chemical driving force (ΔG^γ→α’^), respectively. The fitting results of the tensile test data (Fig. 3a–f) into Eq. (4) for the transformation rate are summarized in Table 3. As the annealing temperature increases, both α (shear-band formation) and β (α’-martensite transformation) parameters increase. As discussed in the previous section, the C and Mn contents decrease while the Al content and austenite grain size increase with increasing annealing temperature. This affects the increase in α parameter and the easy formation of shear bands because of the decreased SFE as well as the increase in β parameter and the easy growth of α’-martensite due to the decreased ΔG^γ→α’^ (which means the increased thermal stability of α’-martensite).

**Table 3:** Values of kinetics parameters (α and β) determined from the transformation data by using Eq. (4).

Specimen	Quasi-static			Dynamic		
	α	β	R^2^	α	β	R^2^
A850	2.72	2.12	0.97	2.68	1.19	0.98
A900	3.22	4.43	0.96	3.12	2.27	0.96
A950	5.35	6.78	0.95	5.42	3.43	0.98

As the strain rate increases from the quasi-static to dynamic loading conditions, the α parameter almost remains, whereas the β parameter largely reduces (Table 3). Since the ‘ε^*^’ is nearly constant with increasing strain rate (Fig. 3), the shear band formation and α’-martensite nucleation are hardly changed with strain rate. According to Talonen *et al*.^22^, the shear band formation is independent of strain rate. Thus, the α’-martensite transformation behavior is mainly affected by the change in the α’-martensite growth behavior closely related with ΔG^γ→α’^ under the dynamic loading.

**Figure 7:**
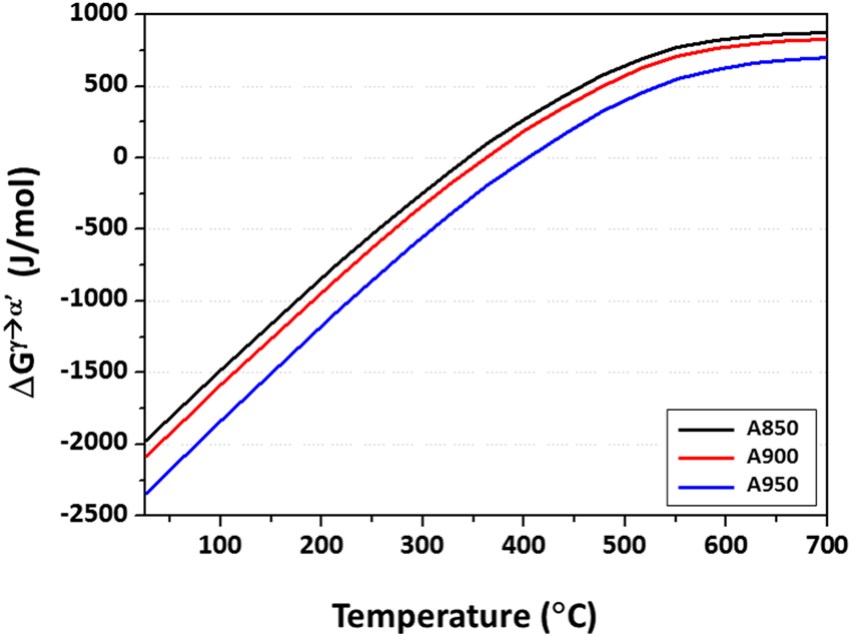
Change of Gibbs free energy between fcc and bcc phases (ΔG^γ→α’^) as a function of temperature for the A850, A900, and A950 specimens. The ΔG^γ→α^’ was thermodynamically calculated by a commercial thermodynamic calculating program, Thermo-CalC with TCFE7 database. The overall ΔG^γ→α^’ decreases with increasing annealing temperature, which indicates the more active TRIP.

In order to analyze the austenite stability change due to the temperature rise under dynamic loading, the difference in ΔG^γ→α’^ at various temperatures was thermodynamically calculated by a commercial thermodynamic calculating program, Thermo-CalC with TCFE7 database, and the results are shown in Fig. 7. Before the thermodynamic calculation, the temperature rising occurring during the dynamic tensile test was calculated by themo-elasto-plastic finite element method (FEM) analyses, and detail results were described in following section. The overall ΔG^γ→α^’ decreases with increasing annealing temperature, which indicates the more active TRIP. As the temperature increases from room temperature to 125, 172, and 145 °C in the A850, A900, and A950 specimens, respectively, the increased amounts of ΔG^γ→α’^ are 653.2, 986.4, and 787.5 J/mol, respectively. Under the dynamic loading, the increase in ΔG^γ→α’^ implies that the chemical driving force for the γ→α’ transformation decreases. The increase in ΔG^γ→α’^ reveals that the decrease in transformation rate is mainly attributed to the reduced thermal stability of α’-martensite because the shear band formation is hardly changed. The M_S_ is highest in the A950 specimen whose austenite fraction and grain size are largest, which means that the thermal stability of austenite is lowest. The austenite fraction reduced rapidly by martensitic transformation in A950 specimen, and A950 specimen shows low ductility as a result of lack of additional TRIP under the quasi-static loading. Under the dynamic loading, however, the thermal stability of austenite increases by temperature rise, the TRIP occurs in an appropriate rate, which promotes the continuous strain hardening and consequently excellent strength-ductility combination. This result is confirmed by the formation of more α’-martensite under the dynamic loading than under the quasi-static loading (Fig. 6(a,b)).

### Effects of temperature rise on austenite stability

The temperature rise occurring during the tensile test is attributed to the heat evolved from plastic deformation and phase transformation. Since the experimental measurement of temperature rise in the dynamic tensile test is very difficult, the temperature distributions inside the dynamically tensioned specimens were calculated by thermo-elasto-plastic finite element method (FEM) analyses using commercial FEM codes of ABAQUS/Standard and ABAQUS/Explicit for quasi-static and dynamic solutions, respectively. Loading conditions were the same as experimental ones, as described in the method section. Modelling of a half of the specimen and split Hopkinson tensile bar is sufficient to simulate the whole system because of the mirror symmetry. Reduced cubic element with thermal information (C3D8RT) was selected to simulate both mechanical and thermal effects. Element numbers were 1,900 for an incident bar, 630 for a striker bar, 6,337 for a transmission bar, and 67,200 for the specimen. Thermal conductivity, specific heat, and inelastic heat fraction were set to be 54 W/mK^44^, 0.49 kJ/kgK^45^, and 0.9, respectively. Johnson-Cook (JC) constitutive model^46^, which is suitable to simulate high strain rates and high-temperature deformation of metals, expresses the stress as a function of plastic strain. The JC model is given by:

5 $$\bar{\sigma }=({\rm{A}}+{\rm{B}}\,{(\bar{{\varepsilon }})}^{{\rm{n}}})\,(1+{\rm{C}}\,\mathrm{ln}\,\dot{\bar{{\varepsilon }}})\,(1-{({{\rm{T}}}^{\ast })}^{{\rm{m}}})$$

where A, B, n, C, and m are the yield stress at reference temperature and reference strain rate, coefficient of strain hardening, strain hardening exponent, strain rate hardening coefficient, and thermal softening exponent, respectively. $\bar{\varepsilon }$ is the equivalent plastic strain, $\dot{\bar{\varepsilon }}$ is the plastic strain rate, and T* is the homologous temperature defined as:

6 $${{\rm{T}}}^{\ast }=({\rm{T}}-{{\rm{T}}}_{{\rm{r}}})/({{\rm{T}}}_{{\rm{m}}}-{{\rm{T}}}_{{\rm{r}}})$$

where T, T_r_, and T_m_ are the current temperature, reference temperature (usually room temperature), and melting temperature, respectively. A, B, n, and C were calculated from the experimental results, and m was obtained by fitting the stress-strain curve. The JC model parameters are listed in Table 4.

**Table 4:** Values of parameters for Johnson-Cook model.

Specimen	A	B	n	C	m
A850	572	1150	0.80		
A900	489	1284	0.76	0.0158	1.4
A950	411	2013	0.88		

**Figure 8:**
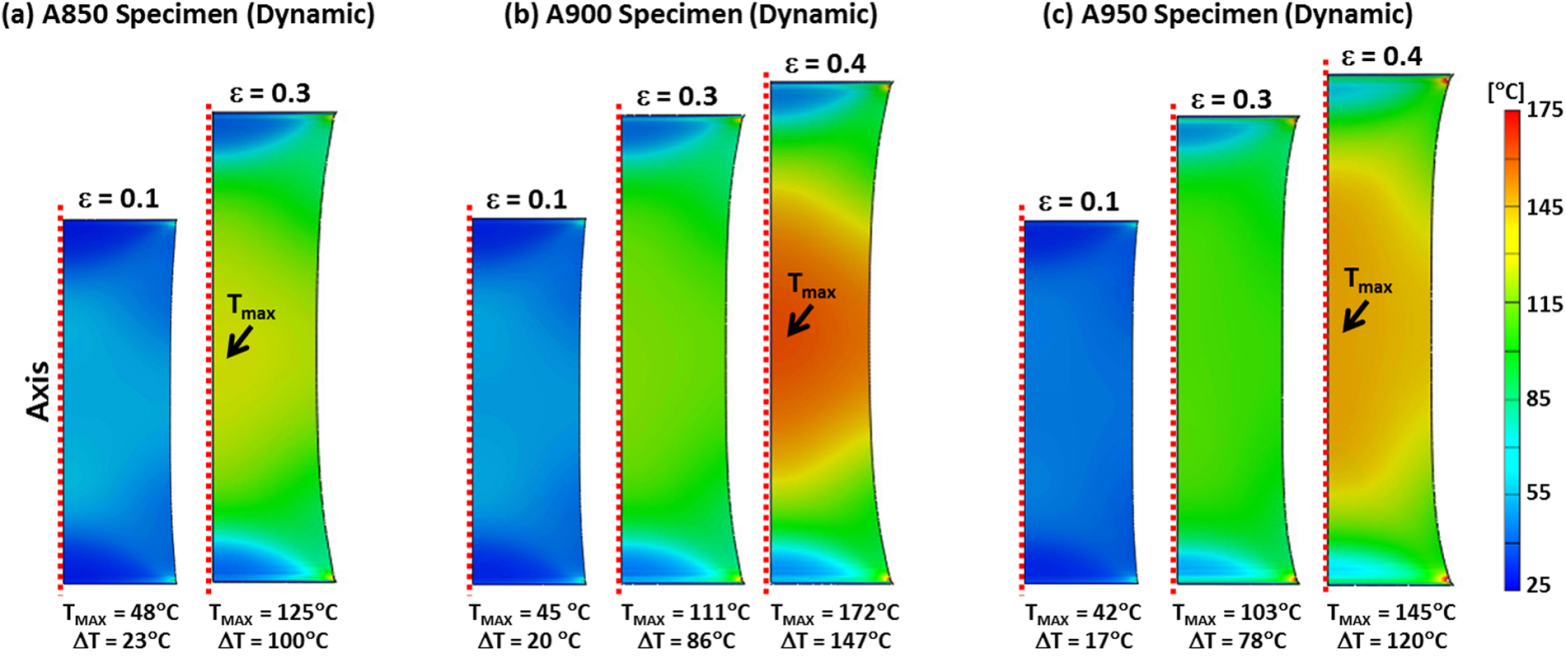
Temperature distributions inside the dynamically tensioned specimens calculated by finite element method (FEM) analysis in the true strain range of 0.1~0.4 for the (**a**) A850, (**b**) A900, and (**c**) A950 specimens.

Figure 8(a–c) shows temperature distributions of the dynamically tensioned specimens at the true strain range of 0.1~0.4. The temperature tends to increases homogeneously up to the true strain of 0.3 in all the specimens. The temperature localization at the gage center occurs more seriously in the A900 specimen than in the A950 specimen (Fig. 8(b,c)), although the uniform elongations of the A900 and A950 specimens are almost same (about 0.42) (Fig. 3(e,f)). This might be attributed to the high strain-hardening rate of the A950 specimen.

Under the dynamic loading, the tensile deformation energy transfers to the interior thermal energy to induce the temperature rise, and this temperature rise is dependent on the thermal energy varied with the strengths of the annealed specimens. Since the time for the interior heat to be emitted outside is not enough, the temperature rise is concentrated into the specimen center region. The temperature rise increases rapidly as the strain increases under the dynamic loading in all the annealed specimens. Particularly in the A850 specimen, the temperature rise at the gage center reaches 125 °C at the true strain of 0.3 (Fig. 8(a)). The martensitic transformation rapidly occurs from the true strain of 0.3 (ε*) under the quasi-static loading (Fig. 3(a)), and thus the A850 specimen is sufficiently elongated as the necking is delayed by the TRIP mechanism (Fig. 2(a)). On the contrary, under the dynamic loading, the increased temperature at the true strain of 0.3 (Fig. 8(a)) leads to the specimen failure as the martensitic transformation hardly occurs (Fig. 3(d)). In the A900 specimen, the TRIP occurs (Fig. 3(e)) because the austenite stability and temperature rise are smaller at the true strain of 0.3 than those of the A850 specimen (Fig. 8(b)). However, the TRIP amount under the dynamic loading is smaller than that under the quasi-static loading because of the increase in austenite stability due to temperature rise, which results in the lower strain-hardening rate and consequent failure at the lower strain than the quasi-static loading. The A950 specimen shows the high strain-hardening rate and the failure without plastic instability as the rapid TRIP works actively from the early deformation stage under the quasi-static loading (Fig. 3c). Under the dynamic loading, the ductility is largely increased by the more martensitic transformation during the deformation (Fig. 3f) because the austenite stability might be optimized by the temperature rise.

It is also noted that deformation twins are formed in the A850 and A900 specimens under the quasi-static loading (Figs 4(b) and 5(b)), whereas they are not formed under the dynamic loading (Figs 4(c) and 5(c)). This TWIP behavior can be readily explained by the SFE change from the temperature rise. At the strain of 0.3 where twins are not found in the A850 and A900 specimens under the dynamic loading, the SFEs calculated from Eq. (3) in consideration of the temperature rise are 73.2 and 69.0 mJ/m^2^, respectively. Since these SFEs lie far above the deformation twining SFE range, the twinning does not occur under the dynamic loading^17,20,30^.

The present study on microstructural evolution related with deformation mechanisms such as TRIP and TWIP would give a good way for plausibly explaining effects of austenite stability on quasi-static and dynamic tensile properties in duplex lightweight steels. It is importantly noted that the martensitic transformation occurs homogeneously and continuously during the whole deformation to obtain excellent tensile properties. The austenite stability is varied with composition, temperature, grain size, and grain orientation. The transformation of all austenite to martensite until the failure is idealistic, but it is almost impossible because the amount of austenite for the TRIP is limited and the control of austenite stability is very difficult. Thus, the occurrence of TRIP at a maximum number of austenite grains during the deformation is most favorable for achieving excellent tensile properties. In the present three specimens, the transformation rate decreases with increasing strain rate, although effects of strain rate on total amount of martensitic transformation are different (Fig. 3(a–c) *vs*. Figure 3(d–f)). When the transformation rate is too rapid, the stress is concentrated at the transformed martensite and its interfacial boundary, which easily induces the cleavage fracture^28^. In the case of the slow transformation rate, mobile dislocations generated from the transformed martensite can readily move to adjacent phases, and void nucleation is activated by the homogeneous deformation between phases, which induces the ductile fracture.

Under the dynamic loading, the temperature rise due to adiabatic heating raises the austenite stability, thereby delaying TRIP or TRIP-TWIP transition. In the A850 specimen, the suppression of TRIP and TWIP leads to the insufficient strain hardening and low ductility. In the A900 specimen, the TRIP occurs, but its low fraction results in the insufficient strain hardening and consequent decrease in ductility. In the A950 specimen whose austenite stability is lowest, the very low ductility is shown by the rapid TRIP under the quasi-static loading. Under the dynamic loading, however, both strength and ductility are improved by an appropriate austenite stability obtained from the temperature rise due to the adiabatic heating. Since the TRIP is closely related with excellent ductility and strain hardening, the achievement of appropriate austenite stability is essentially needed for excellent strength-ductility combination, like in the dynamically tensioned A950 specimen. That is, the TRIP initiates from most of austenite grains having low stability, but should be homogeneous and continuous during the whole deformation to induce the steady strain hardening. Since the austenite stability varied with the temperature rise can change the dynamic deformation behavior, effects of strain rate should be carefully considered in the alloy and process designs of duplex lightweight steels. When genuine dynamic tensile properties are considered, these duplex lightweight steels can be sufficiently applied to automotive steel sheets demanded for stronger vehicle bodies and safety enhancement.

## Conclusions

Quasi-static and dynamic tensile properties of the (α + γ) duplex lightweight Fe-0.3C-8.8Mn-5.1Al steel specimens were investigated in relation with TRIP and TWIP mechanisms.

1. All the annealed specimens exhibited band microstructures of ferrite and austenite elongated along the rolling direction. The austenite volume fraction and grain size of the A850 specimen were 37% and 1.8 μm, respectively, and increased to 44% and 3.0 μm, respectively, in the A950 specimen. According to the M_S_ and SFE calculation results, the austenite stability decreased with increasing annealing temperature.
2. Under the quasi-static loading, the A850 and A900 specimens exhibited the excellent strength and ductility, while the A950 specimen showed the lowest strength and ductility due to the rapid TRIP. The strain-hardening rate curves displayed a multiple-stage strain-hardening behavior, which was attributed to deformation mechanisms of TRIP + TWIP or TRIP. As the annealing temperature increased, the deformation amount required for martensitic transformation decreased, the austenite fraction decreased rather rapidly, and the strain-hardening rate increased. This could be well explained by the reduction in ε*.
3. Under the dynamic loading, the austenite stability tended to increase, while the ε* was not varied much. In the A850 and A900 specimens, the suppression of TRIP and TWIP led to the insufficient strain hardening and low ductility. Although the strain-hardening rate decreased in all three specimens, both strength and ductility were improved in the A950 specimen. The decreased TRIP rate due to the increase in austenite stability promoted the continuous strain hardening and consequently excellent strength-ductility combination.
4. The ε* remained nearly constant in spite of the increased strain rate because the shear band formation and α’-martensite nucleation were hardly changed with the strain rate. According to the thermodynamic calculation of change of Gibbs free energy between fcc and bcc phases (ΔG^γ→α’^), the ΔG^γ→α’^ increment revealed that the decrease in transformation rate was mainly attributed to the reduced thermal stability of α’-martensite because the shear band formation was hardly changed.
5. The increased austenite stability under the dynamic loading was mainly attributed to the temperature rise due to the adiabatic heating. According to the FEM analysis, the temperature rise at the gage center reached 125 °C and 111 °C in the A850 and A900 specimens, respectively, at the true strain of 0.3, which led to the suppression of the TRIP. In the A950 specimen, however, the less rapid TRIP under the dynamic loading induced the largely increased ductility by the more TRIP because the austenite stability might be optimized by the temperature rise. The prevention of TWIP in the A850 and A900 specimens under the dynamic loading was readily explained by the increased SFE to 73.2 and 69.0 mJ/m^2^, respectively.

## Method

### Duplex lightweight steels

The duplex lightweight steel (chemical composition; Fe-0.3C-8.8Mn-5.1Al-(<0.02)(P + S) (wt.%)) was fabricated by a vacuum induction melting. 60-mm-thick plates were homogenized at 1200 °C for 1 hour and hot-rolled at 1100 °C~900 °C to produce 3-mm-thick sheets. Theses sheets were furnace-cooled from 650 °C to simulate a coiling process, cold-rolled to produce 1.4-mm-thick steel sheets, and then annealed at 850, 900, and 950 °C for 10 min in a continuous annealing simulator (model; CAS-AY-II, Ulvac-RIKO, Inc., Japan) to make (α + γ) duplex microstructures. For convenience, the steel sheet specimens annealed at 850, 900, and 950 °C are referred to as “A850”, “A900”, and “A950”, respectively.

### Microstructural analyses

The annealed steel sheet specimens were electro-polished in a 90% CH_3_COOH + 10% HClO_4_ solution at 32 V for the electron back-scatter diffraction (EBSD) observation of longitudinal-short transverse (L-S) plane. The EBSD analysis (step size; 50 nm) was performed by a field emission scanning electron microscope (FE-SEM, Quanta 3D FEG, FEI Company, USA), and the data were interpreted by an orientation imaging microscopy analysis software. Phases of the annealed specimens were identified by X-ray diffraction (XRD, Cu Kα radiation), and volume fractions of phases were measured by using integrated intensities of (200)α and (211)α peaks and (220)γ and (311)γ peaks^47^. Chemical composition maps were obtained by an electron probe micro-analysis (EPMA) microprobe (model; JXA 8530 F, JEOL, Japan). Because of the difficulty in precise measurement of C, the C content was measured by the XRD method^25^.

### Quasi-static and dynamic tensile tests

Plate-shaped tensile specimens (gage length; 30 mm, width; 5 mm, thickness; 1 mm, longitudinal orientation) were tested at strain rate of 10^−3^ s^−1^ at room temperature by a universal testing machine (capacity; 100 kN, model; Instron 8801, Instron Corp., Canton, MA, USA). A split Hopkinson tensile bar was used for dynamic tensile tests^26,48–50^, whose schematic diagram is presented in Supplementary Fig. S3. A plate-shaped tensile specimen (gage length; 7 mm, width; 4 mm, thickness; 1 mm) was prepared in the longitudinal direction. The specimen situated between incident and transmitter bars (diameter; 19 mm) was loaded by a hallow-cylinder-bar-shaped striker bar (outer diameter; 28 mm, inner diameter; 20 mm, length; 700 mm projected at a very high speed using an air pressure of 0.4 MPa (impact velocity; 21 m/s). During the dynamic tensile test, incident, reflective, and transmitted waves were respectively detected at strain gages, and were recorded at an oscilloscope. Among the recorded waves, average tensile strain rate expressed as a function of time was measured from the reflected wave, while tensile stress expressed as a function of time was measured from the transmitted wave. Dynamic tensile stress-strain curves were obtained from these two parameters by eliminating the time term. The strain rate was about 3000 s^-1^. Detailed descriptions of the dynamic test are provided in references^26,48–50^. In order to reduce noises in the data, raw stress-strain curves were smoothened by an adjacent-averaging method^51,52^. The entire tensile tests were performed three times at least for each datum point.

### Digital image correlation (DIC)

DIC technique is a basic method to measure the local deformation strain during the dynamic tensile test^53^. The specimen surface was photographed by using a high-speed camera (model: Phantom V7.3, Komi, Korea), and a vision strain gauge system (model; ARAMIS 5 M, GOM optical measuring techniques, Germany) was used for detecting 3-dimensional coordinates of a deforming specimen surface. White- and black-color lacquers (model: Aqua, Motip Dupli, Germany) were sprayed on the longitudinal-transverse (L-T) plane of the tensile specimen to obtain random black-and-white speckled patterns, which were then used for the strain distribution analysis. The surface was recognized by digital camera images, while coordinates were allocated by pixel images, and deformed surface images were sequentially recorded during the tensile deformation. Nominal and local strains at the center position of specimen gage section were measured along the base line (center line of gage section).

### Data Availability

The data that support the findings of this study are available from the corresponding author upon reasonable request.

## Electronic supplementary material

Supplementary Information
