# Supplementary material for: Interpretation of dynamic tensile behavior by austenite stability in ferrite-austenite duplex lightweight steels
Source: Sci Rep. 2017 Nov 16;7:15726. doi: 10.1038/s41598-017-15991-5 (PMC5691137; doi:10.1038/s41598-017-15991-5)

Supplementary Information

Interpretation of dynamic tensile behavior by austenite stability in ferrite-austenite duplex lightweight steels

Jaeyeong Park [a], Min Cheol Jo [a], Hyeok Jae Jeong [a], Seok Su Sohn [b,*], Jai-Hyun Kwak [c], Hyoung Seop Kim [a], Sunghak Lee [a]

[a] Center for Advanced Aerospace Materials

Pohang University of Science and Technology, Pohang 790-784, Republic of Korea

[b] Max-Planck-Institut für Eisenforschung

Max-Plank-Straβe 1, Düsseldorf 40237, Germany

[c] Sheet Products & Process Research Group

Technical Research Laboratories, POSCO, Kwangyang, 545-090, Republic of Korea

*Corresponding author: S.S. Sohn

bbosil7@postech.ac.kr

Tel: +82-54-279-8654

Fax: +82-54-279-5887

Supplementary Figure S1. Profiles of incident, reflected and transmitted waves obtained from the split Hopkinson tensile bar for the (a) A850, (b) A900, and (c) A950 specimens, from which dynamic stress-strain curves were drawn.

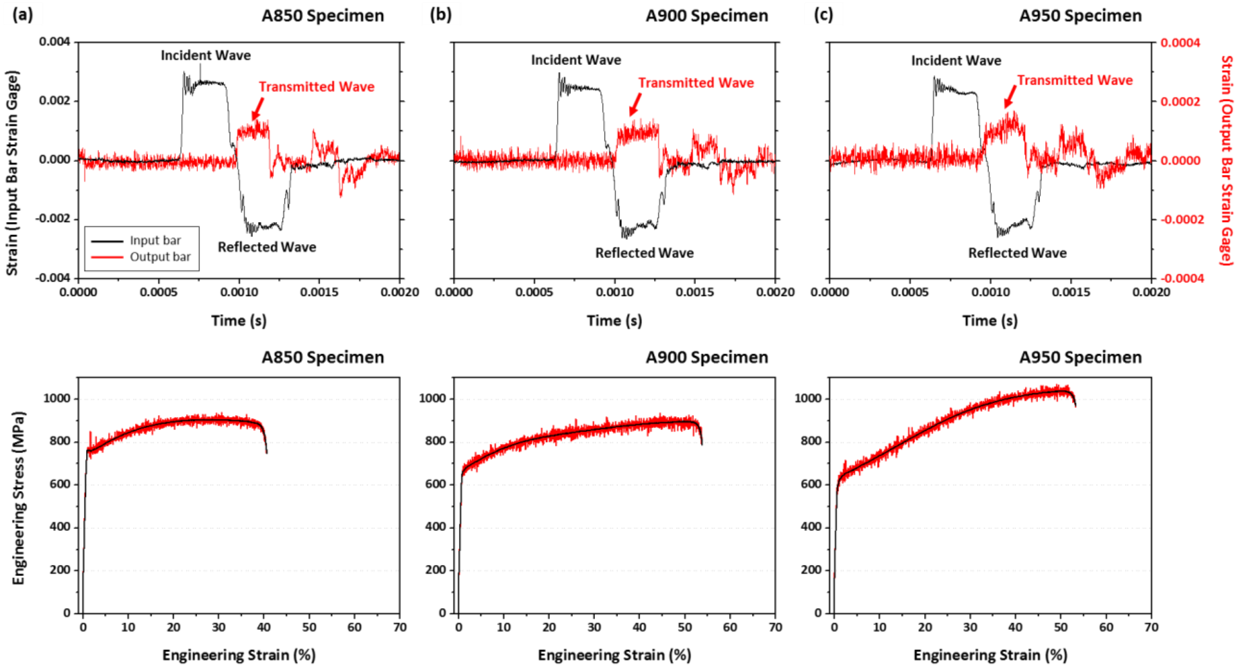

Supplementary Figure S2. (a) Digital images of strain distribution and (b) strain distribution curves of the dynamically tensioned A850 specimen. Digital images are numbered by 'D_1' through 'D_8', which are also marked by arrows in the dynamic stress-strain curve of Fig. 2a. Local strain distribution curves are almost flat until the deformation proceeds to the D_5 stage, which shows the uniform deformation behavior. The red arrows indicate the region of plastic instability.

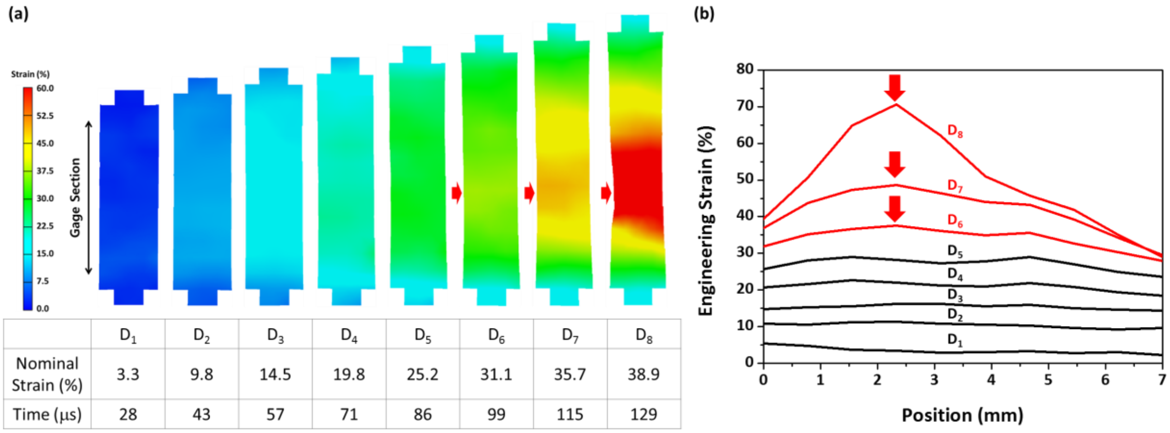

	D_1	D_2	D_3	D_4	D_5	D_6	D_7	D_8
Nominal Strain (%)	3.3	9.8	14.5	19.8	25.2	31.1	35.7	38.9
Time (μs)	28	43	57	71	86	99	115	129

Supplementary Figure S3. Schematic diagram of the Split Hopkinson Bar. A plate-shaped tensile specimen (gage length; 7 mm, width; 4 mm, thickness; 1 mm) was prepared in the longitudinal direction. The specimen situated between incident and transmitter bars (diameter; 19 mm) was loaded by a hallow-cylinder-bar-shaped striker bar (outer diameter; 28 mm, inner diameter; 20 mm, length; 700 mm projected at a very high speed using an air pressure of 0.4 MPa (impact velocity; 21 m/s). During the dynamic tensile test, incident, reflective, and transmitted waves were respectively detected at strain gages, and were recorded at an oscilloscope. Among the recorded waves, average tensile strain rate expressed as a function of time was measured from the reflected wave, while tensile stress expressed as a function of time was measured from the transmitted wave. Dynamic tensile stress-strain curves were obtained from these two parameters by eliminating the time term. The strain rate was about 3000 s^{-1}.

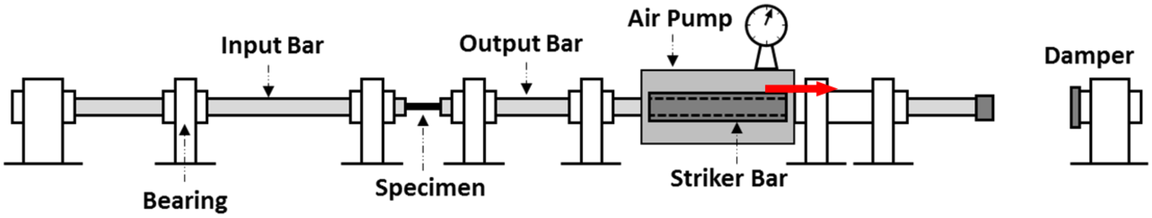

Supplement: Supplementary file 1 — Supplementary Information [file 41598_2017_15991_MOESM1_ESM.pdf]
